# Dynamic Regulation of the Light-Harvesting System through State Transitions in Land Plants and Green Algae

**DOI:** 10.3390/plants12051173

**Published:** 2023-03-03

**Authors:** Hui Shang, Mei Li, Xiaowei Pan

**Affiliations:** 1 College of Life Science, Capital Normal University, Beijing 100048, China; 2 National Laboratory of Biomacromolecules, CAS Center for Excellence in Biomacromolecules, Institute of Biophysics, Chinese Academy of Sciences, Beijing 100101, China

**Keywords:** photosynthesis, state transitions, light-harvesting complex, photosystem I, structure

## Abstract

Photosynthesis constitutes the only known natural process that captures the solar energy to convert carbon dioxide and water into biomass. The primary reactions of photosynthesis are catalyzed by the photosystem II (PSII) and photosystem I (PSI) complexes. Both photosystems associate with antennae complexes whose main function is to increase the light-harvesting capability of the core. In order to maintain optimal photosynthetic activity under a constantly changing natural light environment, plants and green algae regulate the absorbed photo-excitation energy between PSI and PSII through processes known as state transitions. State transitions represent a short-term light adaptation mechanism for balancing the energy distribution between the two photosystems by relocating light-harvesting complex II (LHCII) proteins. The preferential excitation of PSII (state 2) results in the activation of a chloroplast kinase which in turn phosphorylates LHCII, a process followed by the release of phosphorylated LHCII from PSII and its migration to PSI, thus forming the PSI–LHCI–LHCII supercomplex. The process is reversible, as LHCII is dephosphorylated and returns to PSII under the preferential excitation of PSI. In recent years, high-resolution structures of the PSI–LHCI–LHCII supercomplex from plants and green algae were reported. These structural data provide detailed information on the interacting patterns of phosphorylated LHCII with PSI and on the pigment arrangement in the supercomplex, which is critical for constructing the excitation energy transfer pathways and for a deeper understanding of the molecular mechanism of state transitions progress. In this review, we focus on the structural data of the state 2 supercomplex from plants and green algae and discuss the current state of knowledge concerning the interactions between antenna and the PSI core and the potential energy transfer pathways in these supercomplexes.

## 1. Introduction

Oxygenic photosynthesis is one of the most important biological processes and sustains almost all life on Earth. Light capture and subsequent charge separation occur in photosystem I (PSI) and photosystem II (PSII). In oxyphototroph organisms of the green lineage such as green algae and land plants, both PSI and PSII are multi-subunit pigment-protein supercomplexes consisting of a core complex and outer light-harvesting complexes (LHCs) [1,2]. LHCIs with apo-proteins encoded by the *lhca* genes are associated with the PSI core, forming the PSI–LHCI complex. LHCIIs possessing apo-proteins encoded by *lhcb* genes are mainly attached to the PSII core, constituting the PSII–LHCII complex [3,4,5]. All these LHC proteins are similar in overall folding and bind large numbers of chlorophyll and carotenoid molecules, greatly increasing the absorption cross section of the two photosystems. While LHCIs usually form heterodimers, LHCIIs exist as trimeric and monomeric forms, known as the major and the minor LHCIIs [4,6].

**Figure 1:**
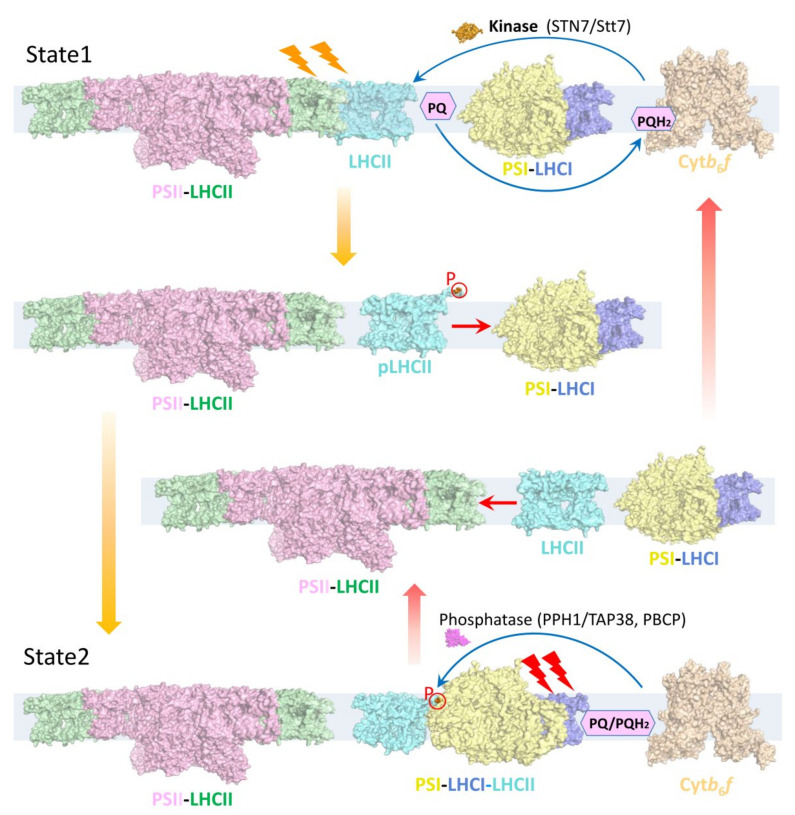
Model of state transitions in the thylakoid membrane. During state transitions, LHCII is reversibly phosphorylated; the dephosphorylated and phosphorylated forms bind to PSII (state 1) and PSI (state 2), respectively. When PSII is preferentially excited, PQ molecules are reduced to PQH_2_. Docking of PQH_2_ to cytochrome *b*_6_*f* (Cyt*b*_6_*f*) leads to the activation of a chloroplast kinase (STN7/Stt7), which is required for the phosphorylation of LHCIIs, causing phosphorylated LHCIIs (pLHCII, cyan) to dissociate from PSII and associate with PSI-LHCI, forming the PSI–LHCI–LHCII supercomplex (state 2). When PSI is preferentially excited, PQH_2_ is oxidized to PQ, and phosphatases (PPH1/TAP38 in plants and PPH1 and PBCP in *C. reinhardtii*) catalyze the dephosphorylation of mobile LHCIIs and their re-association with PSII (state 1). Coordinates (PDB codes) used for the structural representation are 5XNM (PSII–LHCII), 4XK8 (PSI-LHCI), 5ZJI (PSI–LHCI–LHCII and pLHCII), 1RWT (LHCII), 6RQF (Cyt*b*_6_*f*), 4IX3 (Stt7), 4YZG (PPH1).

The antenna systems of PSI and PSII are composed of proteins bound to different pigments. As a result, PSI responds best to far-red light, while PSII is optimized for capturing red light [7,8]; thus, their excitation may be imbalanced in the natural environment in which light quality and quantity vary. When PSII is preferentially excited (state 2), plastoquinone (PQ) is reduced to plastoquinol (PQH_2_) on docking to the cytochrome *b*_6_*f* complex, then a chloroplast kinase (STN7 in higher plants and its orthologue Stt7 in *Chlamydomonas reinhardtii* (*C. reinhardtii*)) is activated and subsequently phosphorylates the major LHCII trimers [9,10]. This event is followed by the release of phosphorylated LHCII trimers from the PSII and their migration towards PSI, where they form the PSI–LHCI–LHCII supercomplex (also termed state 2 supercomplex). This process is reversible, as the preferential excitation of PSI (state 1) induces the dephosphorylation of LHCIIs by phosphatases (TAP38/PPH1 in plants and PPH1 and PBCP in *C. reinhardtii*) and the re-association of LHCII trimers with PSII [11,12,13] (Figure 1). This process of balancing the light excitation energy between the two photosystems is called state transitions, a short-term light acclimation mechanism [8,14]. The process of state transitions has been widely studied in green algae and land plants, and green algae exhibit a stronger amplitude of state transitions than higher plants [15]. State transitions were previously supposed to be correlated with cyclic electron flow (CEF) in *C. reinhardtii* [16], because both processes are regulated by the redox state in chloroplasts, whereas later it was argued that the two processes are independent but usually coexist [17]. State transitions were reported to involve the remodeling of thylakoid membranes ultrastructure [18,19], and the detached LHCIIs may also be converted into an energy-quenching form, to avoid photodamage [20,21,22]. While a previously reported electron microscopic projection map of plant PSI–LHCI–LHCII revealed that one LHCII trimer binds to the PSI core [23,24], a number of studies indicated that PSI in higher plants binds more than one LHCII trimer, and PSI located in the grana margins has a larger antenna size probably due to the binding of extra LHCIIs [25,26,27]. Recent studies reported that in addition to phosphorylated LHCII, additional unphosphorylated LHCIIs are able to associate with plant PSI on other sites under state 2 conditions, and all LHCIIs are able to transfer the excited energy to the PSI core [28,29,30].

The structures of kinases and phosphatases involved in the state transitions process were solved through an X-ray crystallographic method [31,32]; however, high-resolution structural information on the state 2 complex is lacking due to the difficulties in obtaining well-diffracted crystals of large supercomplexes. It is not clear how phosphorylated LHCII recognizes and stably binds to PSI under state 2 conditions and why the dephosphorylation of LHCII results in its dissociation from PSI under state 1 conditions. In recent years, given the rapid development of cryogenic electron microcopy (cryo-EM), a large number of structural data on photosynthetic supercomplexes have been accumulated. High-resolution cryo-EM structures of the state 2 supercomplex from higher plants and green algae have been reported [33,34,35]. In addition, a large PSI complex was reported recently from moss *Physcomitrium patens* (*P. patens*) (*Pp*PSI-L) that includes a phosphorylated LHCII trimer (Available online: https://doi.org/10.1101/2023.01.18.524345, accessed on 20 January 2023). In the following sections, we will focus on recent research advances in the structures of state 2 supercomplexes and review their overall architecture, the similarities and the differences between supercomplexes from various species, the assembly details regarding antennae and the PSI core, the arrangement of pigments and potential energy transfer pathways.

## 2. Highly Conserved Core Subunits and Variable LHCIs Constitute the PSI–LHCI Complex in Land Plants and Green Algae

**Figure 2:**
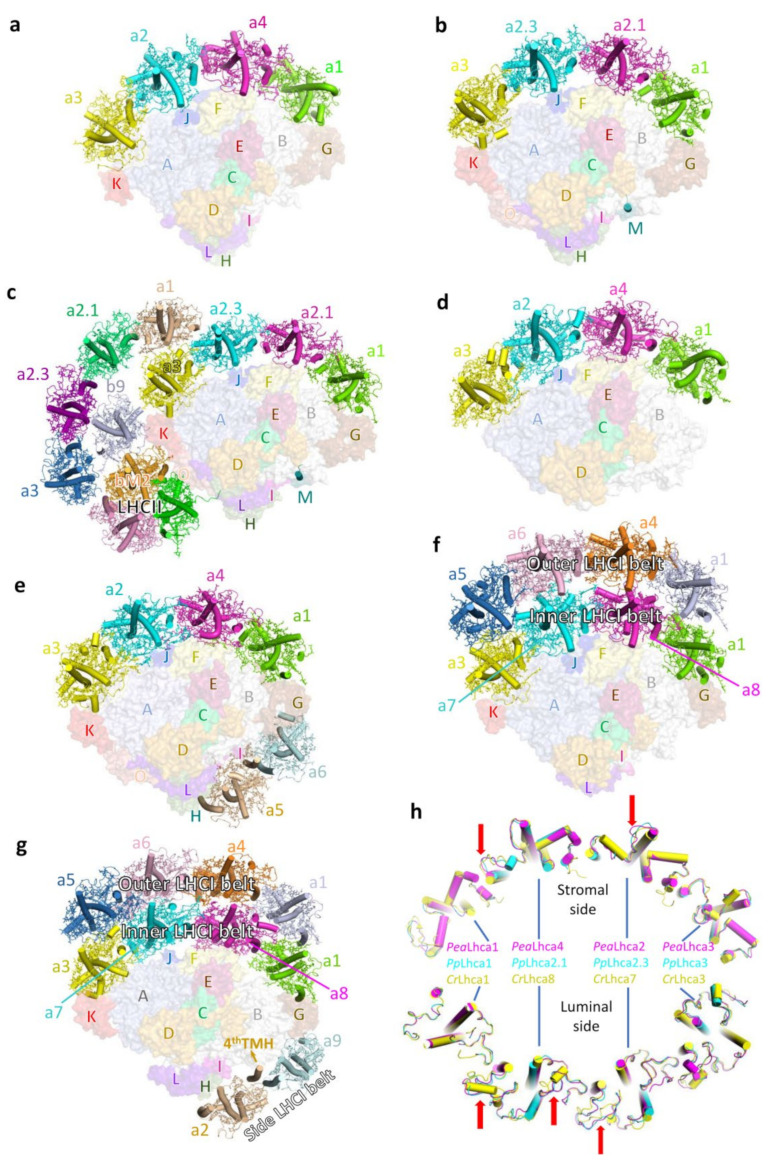
Overall structure of PSI–LHCI complexes from land plants and green algae. (**a**), Structure of the pea PSI–LHCI (PDB: 4Y28). (**b**), Structure of *Pp*PSI-S (PDB: 7KSQ). (**c**), Structure of *Pp*PSI-L (PDB: 8HTU). (**d**), Structure of *D. salina* PSI–LHCI with 4 Lhca proteins (PDB: 6RHZ). (**e**), Structure of *D. salina* PSI-LHCI with 6 Lhca proteins (PDB: 6SL5). (**f**), Structure of *Cr*PSI-LHCI with 8 Lhca proteins (PDB: 6JO6). (**g**), Structure of *Cr*PSI-LHCI with 10 Lhca proteins (PDB: 6IJO). The Lhca proteins are displayed as a cartoon, and their pigments, lipids and other co-factors are shown as sticks. The core subunits are displayed as a surface in different colors and labeled with a single letter (for example, the letter A represents PsaA). *Pp*PsaM in (**b**,**c**) is highlighted. (**h**), Comparison of the Lhca proteins in the (inner) LHCI belt from pea, *P. patens* and *C. reinhardtii* viewed from the stromal side and the luminal, side respectively. Lhca proteins from *P. patens* and *C. reinhardtii* were superposed separately on pea Lhca in their corresponding positions. Larger structural differences are highlighted by red arrows.

Multiple structures of PSI–LHCI from green lineages have been reported, including PSI–LHCI from land plants *Pisum sativum* (Pea) [36,37,38] and moss *P. patens* [39,40] and from the green algae *C. reinhardtii* [41,42], *Bryopsis corticulans* (*B. corticulans*) [43] and *Dunalliela salina* (*D. salina*) [44,45] (Figure 2). The core structures of PSI–LHCI from these phototrophs are highly conserved, containing 13–15 subunits (PsaA–PsaO). One of these subunits, called PsaM, was lost from the genome of flowering plants as well as of the green algae *C. reinhardtii* and *D. salina*, but has been retained in both the green alga *B. corticulans* and the moss *P. patens*. In addition, while the PsaN-encoding gene is still present in flowering plants and green algae, it was lost in mosses. However, PsaN is absent in all PSI–LHCI structures, presumably due to its loose association with other subunits.

The LHCIs in green lineage organisms are variable, presumably as a result of adaptation to their different ecological niches. Flowering plants contain six Lhca proteins (Lhca1–6) [46], four of which (Lhca1–4) stably bind the PSI core. These four Lhca proteins are arranged as a semispherical belt and are composed of two heterodimers, Lhca1–Lhca4 and Lhca2–Lhca3 [36,47]. Lhca2 and Lhca4 are located in the middle of the LHCI belt, and Lhca3 and Lhca1 are located at the two edges, where they closely interact with the PsaK/PsaA and PsaG/PsaB subunits from the PSI core, respectively (Figure 2a) [36,38]. A larger PSI binding two additional Lhcas (probably, the Lhca1–Lhca4 dimer) was previously revealed by an EM projection map [48]. However, a high-resolution structure that can provide more detailed information regarding the subunit composition and assembly pattern remains to be solved. Lhca5 and Lhca6 are expressed only at sub-stoichiometric levels [46]. These two Lhca proteins are essential for mediating the interactions between the PSI–LHCI and the type I NADH dehydrogenase-like (NDH) complexes [49,50,51]. In addition, Lhca5 is able to occupy the Lhca4 position in the Arabidopsis mutant lacking Lhca4, forming a PSI–LHCI complex that resembles that found in wild-type plants [52,53,54].

Bryophytes (i.e., liverworts, mosses, hornworts), which belong to the green lineage, diverged from the ancestor of seed plants and represent the intermediate plant form that transited from aquatic to terrestrial life [55,56]. One of these bryophytes, namely, *P. patens,* is a model organism of mosses used to study the biology of early land plants [56]. *P. patens* contains only four LHCIs (Lhca1–3 and Lhca5) compared to the six found in flowering plants, but its LHC genes are more diverse and redundant than those found in flowering plants. Lhcas1–3 are encoded by several homolog genes, and Lhca5 is encoded by a single gene and shows lower expression level [57,58,59]. *P. patens* possesses several types of PSI complex, and the structures of a small (*Pp*PSI-S) [39,40] and a large PSI (*Pp*PSI-L) (Available online: https://doi.org/10.1101/2023.01.18.524345, accessed on 20 January 2023) were recently solved. PpPSI-S contains an LHCI belt of four Lhca proteins, resembling the PSI–LHCI complex found in vascular plants (Figure 2b), whereas *Pp*PSI-L binds an additional four-Lhca belt, one Lhcb9 and one LHCII trimer (Figure 2c). The position of Lhca4 in *Pp*PSI-S was previously considered to be occupied by Lhca5 [40], whereas the recent high-resolution cryo-EM structure of *Pp*PSI–LHCI revealed that Lhca2.1 and Lhca2.3 (termed Lhca2b and Lhca2a in the previous structural study) occupy the positions of Lhca4 and Lhca2 in the flowering plant PSI–LHCI, respectively [39]. As a result, the *Pp*PSI–LHCI complex contains an LHCI belt that is organized in a Lhca1–a2.1–a2.3–a3 manner (from PsaG to PsaK) (Figure 2b).

The antenna systems of green algal PSI are more complicated than those of higher plants and possess a high degree of diversity in the structures. For instance, the halotolerant green alga *D. salina* binds four and six LHCI proteins (Figure 2d,e) [44,45]. In comparison, the model unicellular green alga *C. reinhardtii* contains eight and ten LHCI proteins (Figure 2f,g) [41,42], and the macroscopic green alga *B. corticulans* contains ten LHCI proteins [43], forming a PSI–LHCI complex with similar architecture to that of one type of *C. reinhardtii* complexes. The largest green algal PSI–LHCI complex contains three LHCI belts, two of which are termed the inner and the outer belt. These two belts are each composed of four Lhcas and together exhibit a double-crescent shape on the PsaG/F/J/K side of the PSI core. The third LHCI belt (side belt) is composed of two Lhcas, which are attached to the PsaH/I/G side [42,43]. Other types of green algal PSI–LHCI complexes lack the side belt, the outer belt or both (Figure 2d–f). In *C. reinhardtii* PSI–LHCI (*Cr*PSI–LHCI), the inner and outer LHCI belts are composed of Lhca1–a8–a7–a3 and Lhca1–a4–a6–a5, and the side belt comprises Lhca2–Lhca9. Notably, unlike other LHC proteins which possess three transmembrane helices (THM), *Cr*Lhca2 possesses a fourth TMH, which is located close to Lhca9 [41,42] (Figure 2g).

Sequence analysis of Lhca proteins from green algae, mosses and flowering plants showed that Lhca1 and Lhca3 have been highly conserved during evolution, whereas other LHCIs are more distantly related between species. Structural superposing of Lhcas at the level of the (inner) LHCI belt from *Pisum sativum*, *P. patens* and *C. reinhardtii* (PeaLhca1-a4-a2-a3, *Pp*Lhca1-a2.1-a2.3-a3, *Cr*Lhca1-a8-a7-a3) also showed that the two terminal Lhcas (Lhca1 and Lhca3) are almost identical, while the two middle Lhcas exhibit larger conformational variations between green algae and land plants (Figure 2h).

## 3. Similarities and Differences of PSI–LHCI–LHCII Supercomplexes between Land Plants and Green Algae

**Figure 3:**
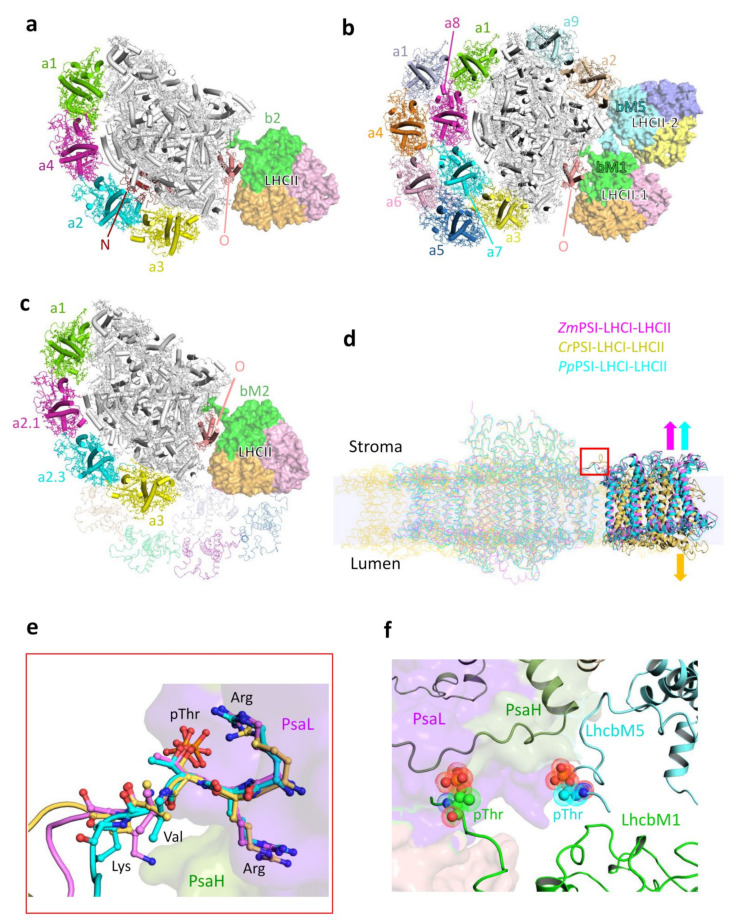
Structures of PSI–LHCI–LHCII from plants and green algae. (**a**), Overall structure of *Zm*PSI–LHCI–LHCII (PDB code: 5ZJI). (**b**), Overall structure of *Cr*PSI–LHCI–LHCII (PDB code: 7DZ7). (**c**), Modeled *Pp*PSI–LHCI–LHCII on the basis of the *Pp*PSI-L structure (PDB code: 8HTU). The PSI–LHCI parts are displayed as a cartoon, with Lhcas, PsaO and *Zm*PsaN shown in different colors and labeled. The other core subunits are shown in white. The LHCII trimers are displayed as a surface and distinguished by different colors. The Lhcb9 and Lhca proteins in the outer LHCI belt from *Pp*PSI-L which are not included in the modeled *Pp*PSI–LHCI–LHCII are shown as a ribbon in (**c**). (**d**), Comparison of the membrane-spanning regions of the three PSI–LHCI–LHCII complexes. LHCIIs (*Cr*LHCII-1) are displayed as a cartoon, and other parts are shown as a ribbon. *Zm*PSI–LHCI–LHCII, *Cr*PSI–LHCI–LHCII and modeled *Pp*PSI–LHCI–LHCII are displayed in magenta, yellow and cyan, respectively. Arrows indicate the deflection of LHCII relative to the membrane. The phosphorylated N-terminal regions of LHCIIs are highlighted in a red box. (**e**), Structural comparison of the first five residues from *Zm*Lhcb2, *Cr*LhcbM1 and *Pp*LhcbM2. (**f**), Zoomed-in view of the binding sites of the N-terminal regions of *Cr*LhcbM1 and *Cr*LhcbM5 in *Cr*PSI–LHCI–LHCII. The phosphorylated Thr residues are highlighted with spheres.

Several types of PSI–LHCI–LHCII supercomplexes have been detected in Arabidopsis, including dimers of PSI–LHCI–LHCII and complexes consisting of PSI–LHCI binding two LHCII trimers (PSI–LHCI–LHCII_2_) in two slightly different positions [30]. Previous biochemical experiments also suggested that under state 2 conditions, plant PSI binds more than one LHCII trimer, albeit with lower affinity [28,29,30]. The additional LHCII trimers are probably in unphosphorylated form and associate with PSI, presumably on the side of the LHCI belt [28]. However, up to now, only the structure of maize PSI–LHCI–LHCII in which one LHCII trimer stably binds to the PSI core (*Zm*PSI–LHCI–LHCII) was solved at a resolution of 3.3 Å [35] (Figure 3a). The PSI–LHCI moiety in the *Zm*PSI–LHCI–LHCII supercomplex was found to be similar to that in the pea PSI–LHCI [36,38]. This moiety contains PsaN and PsaO, neither of which are present in the pea PSI–LHCI structures. PsaN is the only membrane-extrinsic PSI subunit found in the luminal side [60] and easily dissociates from the complex during purification [61]. PsaO is a membrane-embedded subunit that binds to PsaA and provides the docking site for LHCII [35]. When PsaO expression in Arabidopsis is abolished, the level of state transitions significantly decreases [62]. In *Zm*PSI–LHCI–LHCII, one LHCII trimer containing a phosphorylated Lhcb2 is bound to the PSI core on the PsaA side. Lhcb2 possesses a phosphorylated Thr3 in the N-terminal region and interacts directly with PSI core subunits [35]. The structure clearly shows that the phosphorylation of LHCII is critical for the stabilization of PSI–LHCI–LHCII under state 2 conditions.

The state 2 supercomplex identified in the green alga *C. reinhardtii* (*Cr*PSI–LHCI–LHCII) is larger and more intricate than the corresponding complex found in higher plants. The structure of the *Cr*PSI–LHCI–LHCII supercomplex was determined by cryo-EM at resolutions of 3.42 Å [34] and 2.84 Å [33], showing that the supercomplex contains two LHCII trimers (LHCII-1 and LHCII-2) attached to the PSI core. In addition, the two LHCIIs associate with the side belt Lhca2–Lhca9, and together they form a well-connected antenna belt on the opposite side of the double-layered LHCI belt composed of eight Lhcas (Figure 3b). LHCII-1 in *Cr*PSI–LHCI–LHCII (PDB code: 7DZ7) is located in a similar position as the LHCII trimer in the *Zm*PSI–LHCI–LHCII supercomplex, but shows a rotational shift within the membrane plane, suggesting that the interactions between this trimer (LHCII-1 in *C. reinhardtii*) and the PSI core are slightly different between higher plants and green algae. The other LHCII trimer (LHCII-2) is unique to *Cr*PSI–LHCI–LHCII and bridges the LHCII-1 trimer with Lhca2. In the 2.84 Å resolution structure, the two LHCII trimers were identified as LhcbM1, LhcbM2/7 and LhcbM3/M4 in LHCII-1, and as LhcbM5, LhcbM2/7 and LhcbM3/M4 in LHCII-2 (Figure 3b) [33]. Both LhcbM1 and LhcbM5 are phosphorylated at their N-terminal tails, which directly interact with the PSI core [33]. The two LHCII trimers bound to the PSI may explain why the amplitude of state transitions is stronger in *C. reinhardtii* than that in higher plants.

In addition to flowering plants and green algae, state transitions in *P. patens* also involve the phosphorylation of LHCII, although with a lower phosphorylation degree compared with Arabidopsis [58]. The recently reported structure of the *Pp*PSI-L complex (Available online: https://doi.org/10.1101/2023.01.18.524345, accessed on 20 January 2023) revealed that it contains a phosphorylated LHCII trimer, which is located in a position similar to the position of LHCII in the *Zm*PSI–LHCI–LHCII complex. The LHCII monomer, which is phosphorylated at the N-terminal tail and directly interacts with the PSI core, was identified as LhcbM2 (Figure 2c). However, the dissociation of the *Pp*PSI-L complex showed a little effect on the state transitions level of *P. patens* [63,64], indicating that the *Pp*PSI-L supercomplex does not constitute the state 2 complex. Previously, a *P. patens* PSI complex with a molecular mass between those of *Pp*PSI-S and *Pp*PSI-L was isolated and was suggested to correspond to the *P. patens* state 2 complex [64]. An earlier report of low-resolution projection maps of various *Pp*PSI complexes showed that one complex exhibited a similar organization as *Zm*PSI–LHCI–LHCII [58], presumably composed of a plant type PSI–LHCI and one LHCII trimer. Based on these results, we propose that the state 2 complex in *P. patens* is similar to *Zm*PSI–LHCI–LHCII and corresponds to a partial *Pp*PSI-L complex, containing the *Pp*PSI-S moiety plus an LHCII trimer. On the basis of the *Pp*PSI-L structure, we modeled the *P. patens* state 2 complex, in which the LHCII trimer attaches to the PSI on the PsaO side (Figure 3c).

The structures of *Zm*PSI–LHCI–LHCII and *Cr*PSI–LHCI–LHCII and the model of *Pp*PSI–LHCI–LHCII showed that the membrane-spanning regions of PSI–LHCI and LHCII from the three complexes are all not coplanar. LHCIIs in plant PSI–LHCI–LHCII pivot towards the stroma, while LHCII-1 in *Cr*PSI–LHCI–LHCII pivots towards the lumen (Figure 3d). The curved arrangement of these complexes may be related to the changeable thylakoid membrane architecture and be beneficial for the dynamic assembly/disassembly of these complexes under different light conditions.

## 4. Phosphorylated LHCII Is Critical for the Stable PSI–LHCI–LHCII Assembly under State 2 Conditions

Regarding the land plant state 2 complexes, at present, only the structure of the PSI–LHCI–LHCII complex in *Z. mays* is available. However, the previously published low-resolution EM projection map of the Arabidopsis state 2 complex [23,24], together with our modeled *Pp*PSI–LHCI–LHCII show a highly similar organization. These findings clearly indicate that the LHCII trimer in land plant PSI–LHCI–LHCII occupies the same position. In addition, LHCII-1 in *Cr*PSI–LHCI–LHCII is located similarly to LHCII in the land plant supercomplex. All of these LHCIIs contain one monomer (*Zm*Lhcb2, *Cr*LhcbM1 and *Pp*LhcbM2), which is phosphorylated at the N-terminal tail (Figure 3a–c). The N-termini of *Zm*Lhcb2, *Cr*LhcbM1 and *Pp*LhcbM2 possess identical residues (RRtVK) and are well superposed in these structures (Figure 3e). Their N-terminal regions are all rope-shaped and extend to the same surficial pocket of PSI. The residue pThr and the two basic residues immediately preceding pThr form specific interactions with the subunits PsaH and PsaL from the PSI core. A majority of interacting residues from the two core subunits are conserved between plants and green algae. The fact that Lhcb proteins facing PSI–LHCI are all highly similar in terms of structure and sequence and the fact that these Lhcb proteins and PSI exhibit similar interacting patterns indicate that the recognition pattern between the PSI core and the phosphorylated LHCII trimer (LHCII in plants and LHCII-1 in *C. reinhardtii*) is conserved in organisms of the green lineage.

The only PSI–LHCI–LHCII structure solved so far possessing the second LHCII trimer was found in *C. reinhardtii*. LHCII-2 contains a phosphorylated LhcbM5 and associates with PSI via LhcbM5. In comparison with other LhcbM proteins, *Cr*LhcbM5 has an extended N-terminal region, which possesses a phosphorylated Thr33 (pThr33) and interacts widely with the core subunit PsaH and with LhcbM1 from the LHCII-1 trimer (Figure 3f). As a consequence of this structural feature, LhcbM5 not only connects LHCII-2 to PSI, but also stabilizes the binding between LHCII-1 and PSI. Previous reports investigating state transitions in various *C. reinhardtii* LhcbM-deleted mutants established that LhcbM5, but not LhcbM1, is essential for the *Cr*PSI–LHCI–LHCII supercomplex formation and state transitions [33,65]. Residues from PsaH involved in LhcbM5 binding are variable between maize and *P. patens* [33], indicating that the binding site of LHCII-2 is specific for green algae and is absent in plants.

Interestingly, cyanobacteria and red algae exhibit no signs of the state transitions observed in plants and green algae. Consistent with this observation, cyanobacteria and red algae do not contain LHCII and PsaH, and their PsaL lacks the extended stromal loop that is essential for the interaction between LHCII and PsaL found in green lineage [35]. Previous studies showed that state transitions in plants are severely impaired in the absence of the PsaH or PsaL subunit [66]. Together, these results imply that LHCII, PsaH and the extended loop of PsaL co-evolved in the organisms of the green lineage, to allow the association of LHCII with the PSI core and thus the occurrence of state transitions.

Compared to the corresponding LHCII of the PSI–LHCI–LHCII complex of *Z. mays,* LHCII-1 of the *Cr*PSI–LHCI–LHCII complex pivots towards LHCII-2. The two LHCII trimers, together with the Lhca2–Lhca9 heterodimer, form an antenna belt via multiple interactions between adjacent LHCs. Presumably, these close contacts cause the rotational shift of LHCII-1 towards LHCII-2 in the green algal complex [33]. Compared to aquatic algae, land plants face far stronger light intensities and may therefore require a smaller number of antenna proteins than their algae relatives. The loss of the side belt containing Lhca2–a9 may be the main cause of why plants have lost the LHCII-2 trimer. To compensate for this loss, the preserved LHCII-1 then rotates slightly to the PsaK position, as a result of which, the LHCII-1 better associates with the PSI core.

Large supercomplexes usually exhibit flexibility in local regions. Multi-body refinement is an approach to characterize molecular motions in single-particle cryo-EM data [67]. The multi-body refinement of *Cr*PSI–LHCI–LHCII structure at 2.84 Å resolution [33] indicated that two LHCII trimers were mobile relative to PSI as one rigid body, and both lateral and vertical shifts of two LHCIIs were observed in the supercomplex. In addition, we failed to superpose the two LHCII trimers in the two *Cr*PSI–LHCI–LHCII structures solved by different groups when aligned on their PSI core subunits. Together, these observations strongly suggest that the binding of two LHCII trimers with the PSI in the supercomplex is highly dynamic, a feature that may play an essential role in regulating the complex rearrangement and in fine-tuning the excitation energy transfer in the complex.

## 5. Well-Organized Pigment Molecules Constitute Potential Energy Transfer Pathways in PSI–LHCI–LHCII

The state 2 complexes found in *Z. mays*, *C. reinhardtii* and *P. patens* possess numerous chlorophylls (Chls) and carotenoids (Cars), all of which are critical for efficient energy absorption and transfer from the antenna system to the PSI core. In addition, they also play critical roles in the energy-quenching process, primarily in protecting photosystems from high-light damage [68] as well as in stabilizing the supercomplex. The LHCs of the green lineage usually contain 14–17 Chls and 3–5 Cars. Most of the Chl-binding sites, including Chls 603–609 and cluster 610-611-612, and the Car-binding sites L1 and L2 are completely conserved in all LHCs of known structures. Moreover, almost all LHCs contain the Car-binding site N1, and the LHCII trimer possesses the fourth Car-binding site V1 located at the monomer–monomer interface [69,70].

**Figure 4:**
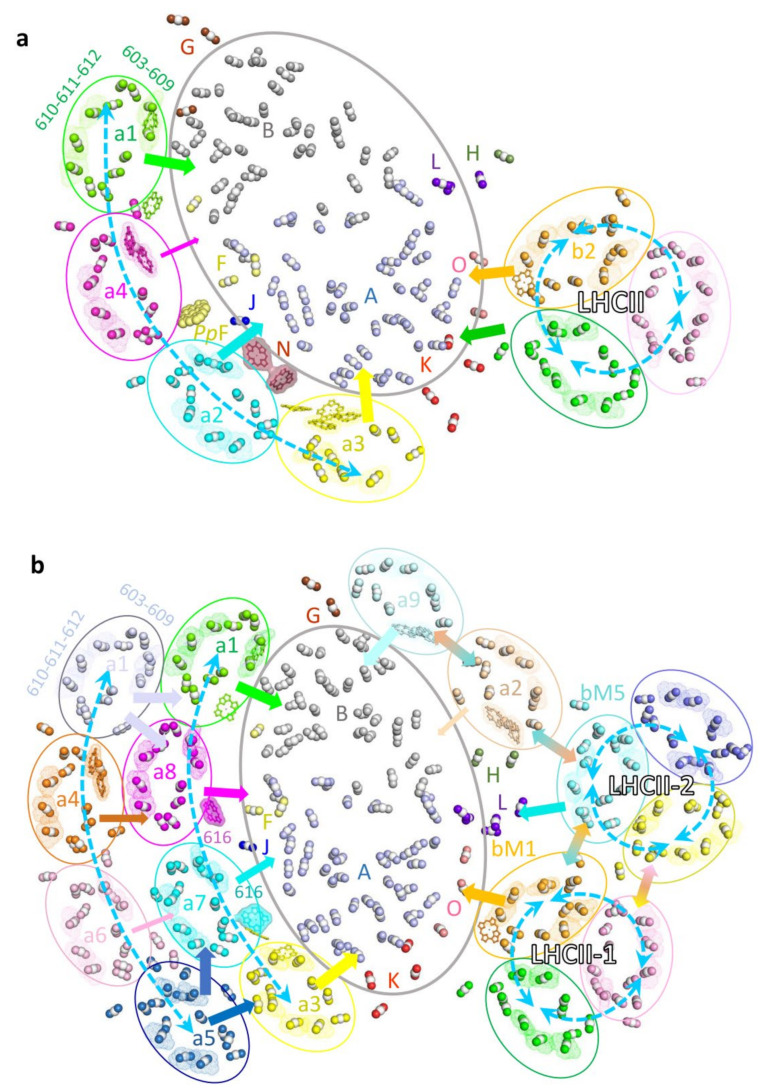
Pigment arrangement and potential energy transfer pathways in the PSI–LHCI–LHCII supercomplexes from land plants and green algae. (**a**), Energy transfer pathways within *Zm*PSI–LHCI–LHCII. (**b**), Energy transfer pathways within *Cr*PSI–LHCI–LHCII. The chlorophyll molecules are simplified with a central Mg and two nitrogen atoms in the sphere mode. All Mg atoms are shown in white, and the nitrogen atoms are shown in the same color as their belonging subunits in Figure 2 and Figure 3. The Chl clusters 610-611-612 and 603-609 in each LHC protein are highlighted with dots. The red Chl pairs in *Zm*Lhca3, *Zm*Lhca4 and *C*rLhca2, *Cr*Lhca4 and *Cr*Lhca9 are shown as sticks and dots. Specific Chls located in the Lhca–core gap region in the *Zm*PSI–LHCI–LHCII and *Cr*PSI–LHCI–LHCII structures, namely, two Chls from *Zm*PsaN and Chls 616 from *Cr*Lhca7 and *Cr*Lhca8, are displayed as surfaces and sticks. One Chl from *Pp*PsaF located between the core and the middle Lhcas in *P. patens* is displayed as spheres. The special Chls which regulate the energy transfer from the antenna to the core on the luminal side are displayed as sticks, including Chls 607 and 617 in Lhca3, Chls 607 and 616 in Lhca1, Chl 614 in *Zm*Lhcb2 and *Cr*LhcbM1.

The LHCII trimers in *Zm*PSI–LHCI–LHCII and *Pp*PSI–LHCI–LHCII, and LHCII-1 in *Cr*PSI–LHCI–LHCII complexes associate with PSI at similar positions. Consequently, these LHCIIs probably deliver the excitation energy to the PSI core via similar pathways (Figure 4). PsaO primarily receives excitation energy from the stromal Chl a611-a612 cluster (the lowest energy state in LHCII) and luminal Chl 614 in the phosphorylated LHCII monomer. In addition, PsaK in plant PSI–LHCI–LHCII might receive the excitation energy from the adjacent LHCII monomer (Figure 4a). In contrast, EET from *Cr*LHCII-1 to PsaK is probably inefficient, since *Cr*LHCII-1, due to its rotational shift, is located more distantly from PsaK compared to LHCII in the plant supercomplex (Figure 4b). The PSI–LHCI–LHCII complex from *C. reinhardtii* contains an additional LHCII (LHCII-2) trimer. In this LHCII, the lowest-energy cluster Chl 611-612 from LhcbM5 is crucial in relaying EET from the LHCII-2 trimer to the core subunit PsaL. In addition, the excitation energy may be equilibrated within the antenna belt composed of two LHCII trimers and the Lhca2–Lhca9 heterodimer through the interfacial Chls, and further transferred to the PSI core (Figure 4b).

The (inner) LHCI belt of three PSI–LHCI–LHCII complexes showed similar architecture and pigment arrangement, suggesting that the pathways used for EET are presumably similar as well. The two terminal Lhca proteins Lhca1 and Lhca3 are conserved from green algae to higher plants and play major roles in EET from the (inner) LHC belt to the core region. The stromal side Chls a603-609(-617 in Lhca3) as well as the luminal side Chl 607 (and Chl 616 in Lhca1) in the two Lhcas are closely connected with Chls from the PSI core. As a result, these Chls are likely to constitute the major EET pathways from the (inner) LHCI belt to the core. Two middle Lhcas vary in different species (a2-a4 in pea and maize, a2.3-a2.1 in *P. patens*, a7-a8 in *C. reinhardtii*), and specific EET pathways from middle Lhcas to the core were observed. For example, the *Zm*PSI–LHCI–LHCII supercomplex contains a luminal PsaN subunit that is located in the gap region between Lhca2 and the PSI core and binds two Chls. As a consequence, the Chls provided by PsaN mediate the EET from Lhca2 to PsaA [35]. The PsaF of *Pp*PSI-S was recently shown to possess an additional Chl located in the stromal gap area between the two middle Lhcas. Because of this additional Chl, specific EET pathways through which the PSI core of *P. patens* accepts excitation energy from both middle Lhca proteins are established [39,40]. When the PSI–LHCI–LHCII complex from higher plant is compared to the *Cr*PSI–LHCI–LHCII complex, it is clearly visible that the two middle Lhcas, *Cr*Lhca7 and *Cr*Lhca8, possess an additional Chl (Chl 616), which is absent in the corresponding Lhcas of plant PSI–LHCI. These Chl 616 molecules function as connectors for efficient EET from the middle Lhcas to the core of *Cr*PSI–LHCI [33,42].

A unique feature of LHCI is the presence of red Chls which absorb long-wavelength light and which represent the lowest energy level within the LHCI [71]. These redshift characteristics are derived from a substitution of the ligation residue (an Asn instead of the usual His) of Chl 603, which is strongly coupled with Chl 609 [72]. The number and position of red Chls in PSI are various among species. For example, Lhca3 and Lhca4 from flowering plants and Lhca3 from mosses carry the red Chls, whereas *C. reinhardtii* lack red Chls in its inner belt, but Lhca4 in the outer belt and Lhca2-Lhca9 in the side belt possess the red Chls. Hence, the (inner) LHCI belt from *C. reinhardtii, P. patens* and plants possess zero, one and two red Chls, respectively. The appearance of more low-energy red Chls in the (inner) LHCI belt may allow a more efficient EET to the core and effective energy quenching in the plant PSI complex; thus, the variation of red Chls may reflect the evolutionary demands of green algae, mosses and flowerer plants adapting to their living environment.

## 6. Conclusions and Perspectives

All photosynthetic organisms face the challenge of continuously adjusting their light capture capabilities to adapt to ever-changing environmental conditions. State transitions constitute a short-term mechanism for light acclimation, necessary to allow the optimal growth and efficient photosynthesis of green algae and plants in their natural environments. In addition to state transitions, several other mechanisms have been developed in plants to regulate their light harvesting and transfer [73], and different protein complexes are involved in these regulatory processes. Structural information on these complexes is crucial for understanding these regulatory mechanisms in photosynthesis. Recent structural analysis of state 2 complexes from green algae and land plants revealed how phosphorylated LHCII recognizes and interacts with the PSI core, thus resulting in a complex formation [33,34,35]. These structures provide detailed information regarding the assembly of antennas, pigment networks and potential energy transfer pathways of these complexes in flowering plants and green algae. In addition, the diverse organizations of the complexes from plants and green algae give clues about the adaptions of photosystems under different light conditions in aquatic and terrestrial environments. However, high-resolution structures of state 2 complexes from other species and state 2 complexes bound with additional LHCIIs in higher plants are still lacking. Specifically, the state 2 complex in moss *P. patens* has not been elucidated, albeit a proposed structural model could be obtained based on the *Pp*PSI-L structure (Available online: https://doi.org/10.1101/2023.01.18.524345, accessed on 20 January 2023). The *Pp*PSI–LHCI–LHCII structure should provide more information for understanding the state transitions in plants. Another critical regulatory mechanism in plants is energy-dependent non-photochemical quenching (qE-NPQ) [74]. Under high light conditions, when the absorbed light energy exceeds the capacity of the photosystems, the excessively absorbed energy is dissipated as heat through qE-NPQ [74]. Two proteins, PsbS and the light-harvesting complex stress-related (LHCSR) protein belonging to the LHC family, are important for inducing the dissipation of excess energy, presumably through interacting with PSII (or PSI) in plants and green algae, respectively [75,76]. While the crystal structure of PsbS was determined [77], LHCSR still awaits structural elucidation in high resolution. In addition, the structure of supercomplexes containing PSII (or PSI) with PsbS or LHCSR is currently lacking due to their unstable combination. PsbS does not bind pigments, thus may be not directly involved in qE quenching. The two acidic residues located in luminal loop regions of PsbS may be able to sense the decreased lumen pH and trigger qE [78], presumably through promoting the rearrangement of the PSII supercomplex in the grana membrane [79]. PsbS was previously suggested to interact with the PSII antenna proteins CP29 and LHCII to activate quenching [80]; however, the exact location of PsbS and its interactions with the PSII antenna remain to be verified. In contrast to PsbS, the LHCSR protein binds both chlorophylls and xanthophylls [81] and was shown to catalyze the quenching of excess energy dissipation in both photosystems in mosses and green algae [82,83]. The structural characterization of photosystems including LHCSR or PsbS will help further elucidate the molecular basis of photoprotective mechanisms in green lineage. The photoprotective mechanism is common to all flowering plants and crops, and previous studies suggested that accelerating the recovery from photoprotection improves photosynthesis and crop productivity [84]. The detailed structural analysis of photosynthetic complexes will provide basic concepts for obtaining plants/crops with increased yield and designing more efficient artificial photosynthetic systems and sustainable energy technologies.

## Data Availability

The data is contained within the manuscript.
